# Enzootic Hepatic Capillariasis (*Calodium hepaticum*) in Street Rats (*Rattus norvegicus*) from Marseille City, France

**DOI:** 10.3390/pathogens9121048

**Published:** 2020-12-14

**Authors:** Cédric Roqueplo, Hubert Lepidi, Hacène Medkour, Younes Laidoudi, Jean-Lou Marié, Bernard Davoust

**Affiliations:** 1 French Military Health Service, 97411 Saint Denis, France; cedric.roqueplo@hotmail.com; 2 Animal Epidemiology Expert Group of the Military Health Service, 37000 Tours, France; jean-lou.marie@wanadoo.fr; 3 IHU-Méditerranée Infection, 13005 Marseille, France; hubert.lepidi@ap-hm.fr (H.L.); hacenevet1990@yahoo.fr (H.M.); younes.laidoudi@yahoo.com (Y.L.); 4 Aix Marseille University, IRD, AP-HM, MEPHI, 13005 Marseille, France; 5 Laboratoire D’anatomo-Pathologie, CHU La Timone, Assistance Publique-Hôpitaux de Marseille, 13005 Marseille, France; 6 Expertise and Defense Health Strategy Division, French Military Health Service, 75000 Paris, France

**Keywords:** *Calodium hepaticum*, *Rattus norvegicus*, rodent, hepatic capillariasis, zoonosis, France

## Abstract

Hepatic capillariasis is a rare and neglected zoonosis affecting wild and synanthropic small rodents. It is caused by infection with *Calodium hepaticum* in liver. Despite the worldwide distribution of the host *Rattus norvegicus* (brown or street rats) in the urban area, the epidemiological status of this parasitosis remains unknown. In the present study, we examined a total of 27 brown rats from the city centre and a garden (four km from the city centre) of Marseille, France. All rats were autopsied and 52% showed the presence of *C. hepaticum* eggs in the liver. This result draws general attention to public health risks, since street rats are living near the human population.

## 1. Introduction

The expansion of urbanisation phenomenon throughout the world leads to an extensive proliferation of rodents within human cities. For example, in Africa, the urban slum population has been doubled in the last 30 years, from 200 to 500 million. It is estimated that after ten years, one billion Africans will be living in urban areas [1]. In cities throughout the world where rodents and humans coexist at high population densities, public health problems, nuisance (degradation of the living environment) and damage caused by these commensal animals are in continuous increase. The brown rat (*Rattus norvegicus*) is a synanthropic species that has dominated urban rodent populations in large parts of the world for many centuries [2]. They live in groups and feed mainly at night on food waste. They are adapted to an environment that allows them to dig burrows in the ground. This species has no predators in French cities; consequently, its proliferation is completely uncontrolled. However, despite their abundance and their invasive characters as well as the risk they cause, brown rats are not the subject of much operational research. Disease control in the brown rats concerns mainly the unapparent zoonotic infections such as leptospirosis [3,4,5].

Marseille is a major Mediterranean port and constitutes the second largest municipality in France with 900,000 inhabitants and it is known to be endemic with street rats. The present study aimed to pathologically explore the circulation of *Calodium hepaticum* in brown rat populations from the centre of Marseille city. 

*Calodium hepaticum* (syn. *Capillaria hepatica*) (Bancroft, 1893) Moravec 1982, belongs to the Trichinelloidea, Capillariidae nematodes [6]. This round worm is a non-segmented and sex-separated individual with a thick cuticle that develops through the successive moults [7]. In addition to the short lifespan of adult forms (an average of two months), their location inside the hepatic parenchyma of the infected hosts explains the difficulty of recovering intact nematodes. Consequently, the morphology of the adult remains incompletely described. Males are about 27 mm long and females about 58 mm long [8]. The worm death occurs spontaneously inside the hepatic parenchyma. This explains why we have only observed fragmented pregnant females or worms in the process of lysis. The eggs measure about 30 × 50 µm and have a polar capsule at each end, these are called bipolar eggs. The lifecycle of *C. hepaticum* is monoxenic [7], starting after the ingestion of embryonated eggs by the definitive host. The first larval stage hatches in the digestive tract (in the small intestine or cecum), crosses the caecal barrier and migrates via the mesenteric and portal hepatic veins towards the liver where all moults occur. It is therefore the macro and microscopic observation of rat livers from the streets of Marseille that led us to highlight this parasitosis, potentially zoonotic.

## 2. Case Details

**Figure 1:**
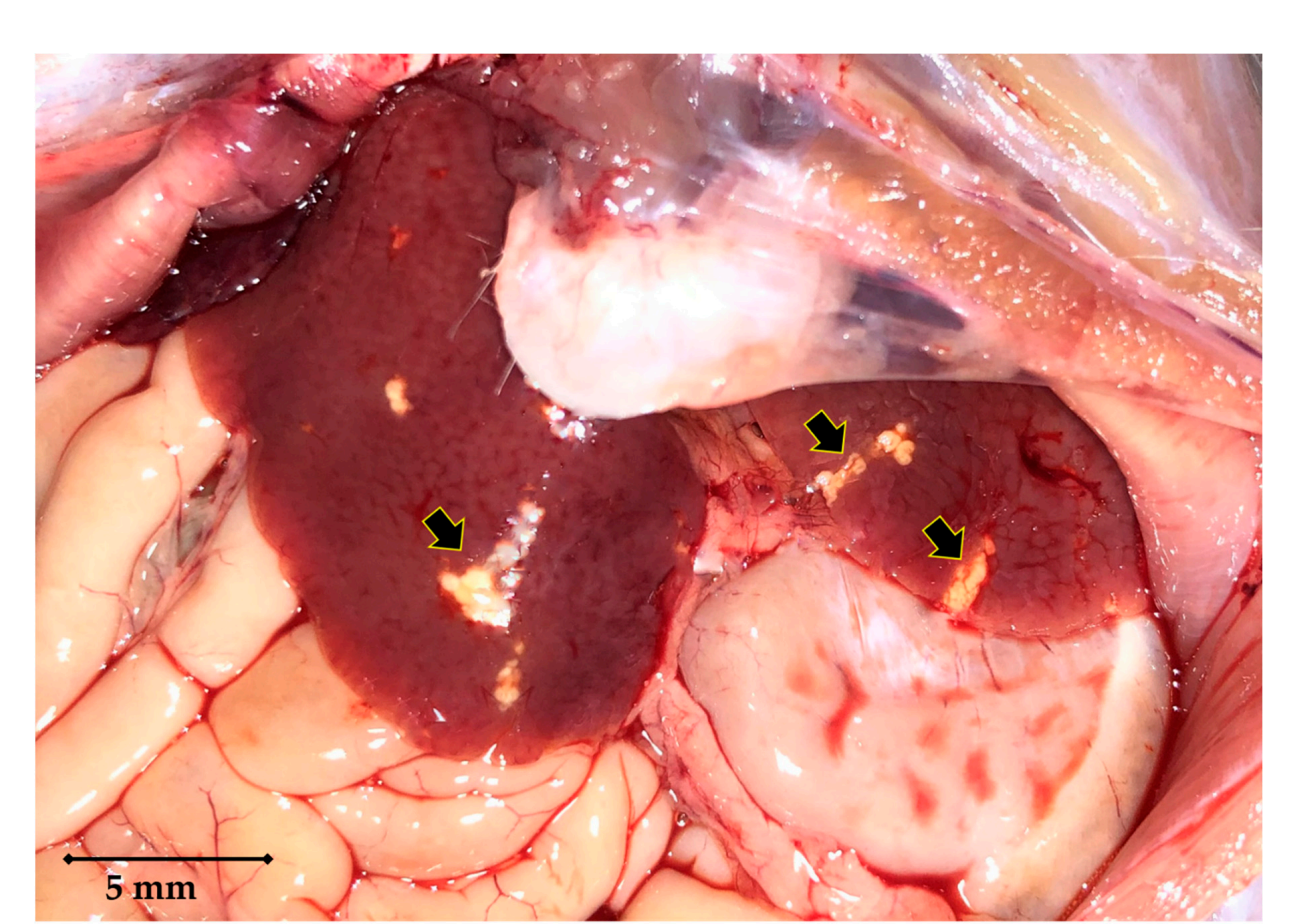
Micrograph showing pathological lesions (arrowed) of liver affected by capillariasis. The yellowish-white lesions are located under the Glisson’s capsule and grouped in patches with irregular forms.

A total of twenty-seven specimens (18 males and 9 females) of *Rattus norvegicus* were studied. Twenty rats were trapped (n = 11) or freshly found dead (accident with a car) (n = 9) in three streets of the city centre of Marseille (43°17′47.2″ N 5°22′47.1″ E; 43°16′34.4″ N 5°23′29.2″ E and 43°17′06.3″ N 5°23′46.6″ E), and seven rats were trapped from a house garden located at 4 km from the centre of Marseille (43°17′44.7″ N 5°25′26.0″ E). The present project was conducted in the framework of a rat control program in compliance with the ethical standards of European regulations governing the care and use of animals in research. According to the Rural Code (Article R214-89) of the French legislation, this project is not considered an experimental procedure and was therefore not subjected to any ethical committee approval in France. Living rats (n = 18) were anaesthetised using ketamine (100 mg/kg) prior to their euthanasia with an overdose of pentobarbital (120 mg/kg). All rats were necropsied, and livers were subjected to pathological examination. All livers were normal in size on visual examination. *C. hepaticum* infection was detected in 14 (8 males and 6 females) out of 27 rats tested, which corresponds to a frequency of infection of 52%. All infested rats were originated from the city centre. *C. hepaticum*-infested livers revealed the presence of yellowish-white spots at the visual examination. These lesions were small (few mm^2^) with irregular contours, grouped in patches which were themselves arranged in no particular order (Figure 1). These spots, located under the Glisson’s capsule, were included in a normal liver parenchyma suggesting the *C. hepaticum* infection.

**Figure 2:**
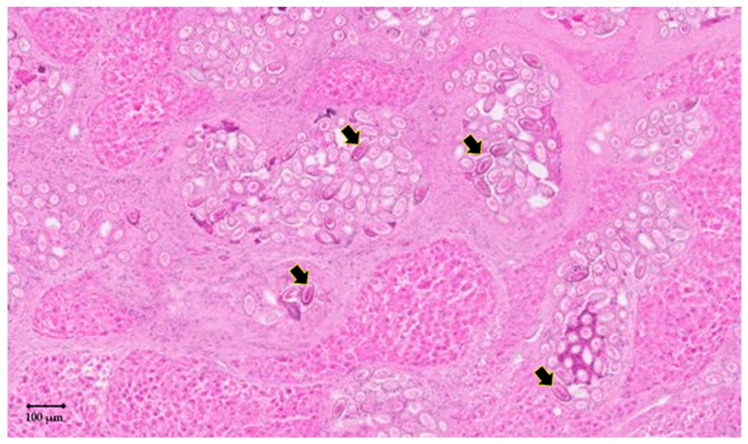
Histologic characteristics of *Calodium hepaticum* infection in rat (*Rattus norvegicus*) liver. Liver parenchyma showed numerous oval or spherical eggs of *C. hepaticum* in a necrotic fibrous tissue (arrow) [haematoxylin–eosin–safran—HES, original magnification ×30].

**Figure 3:**
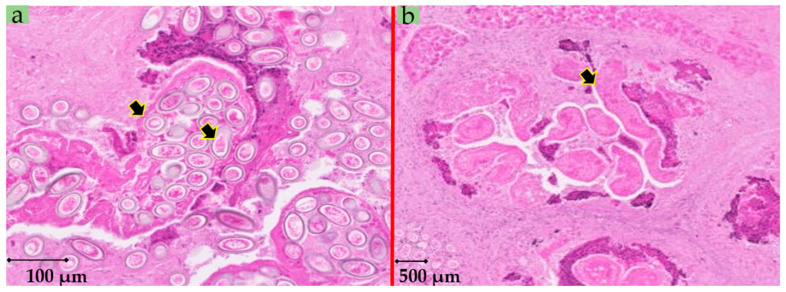
(**a**) Rat liver: uteri of adult worms (*Calodium hepaticum*) with eggs [HES, original magnification ×80]. (**b**) Lysing dead worms [HES, original magnification ×30].

**Figure 4:**
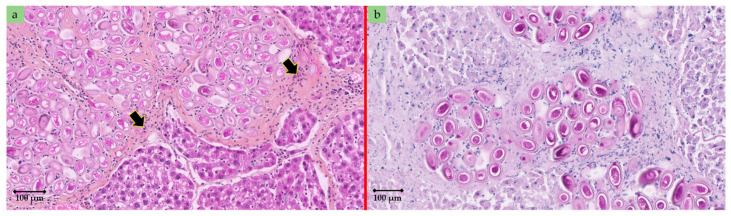
Rat liver. (**a**) eggs of *C. hepaticum* within foci in a fibrous and inflammatory tissue (arrow) [HES, original magnification ×190]. (**b**) eggs of *C. hepaticum* doliiform (barrel-shaped) with thick wall (bilayer) consisting of proteins forming radial striae [Schiff’s periodic acid—PAS, original magnification ×250].

In order to investigate the causative agent of these lesions, a pathological examination was carried out from a section of these patches. Briefly, a 4% buffered formalin solution was used to fix liver tissues. Fixed tissues were then subjected to a serial 3 µm slice sections and were then stained with haematoxylin–eosin–safran (HES) and Schiff’s periodic acid (PAS) prior to microscopic examination. All lesions harboured eggs of *C. hepaticum* (Figure 2). In addition, the uteri of adult worms containing eggs (Figure 3a) and lysing dead worms were also observed (Figure 3b). Within the hepatic parenchyma, eggs were arranged inside small foci of necrosis surrounded by collagenous fibrosis (Figure 4). Moderate mononuclear cell inflammation was demonstrated in this necrotic and fibrous tissue. The eggs are doliiform (barrel-shaped). The egg walls appeared to be thick (bilayer) and consist of proteins forming radial striae visible under the microscope (Figure 4).

## 3. Discussion

Sexually mature females of *C. hepaticum* begin to lay eggs mainly in the liver portal spaces. Once the reproductive function has been fulfilled, the adult parasite dies and gradually disintegrates. The chronic inflammatory reaction of the host gradually encapsulates the eggs in the liver parenchyma [9]. Fibrosis is observed in the liver portal spaces with macrophages and myofibroblasts [10].

The epidemiology of rat hepatic capillariasis is directly related to the life cycle of *C. hepaticum*. This nematode has a direct life cycle without an intermediate host. However, it requires two hosts to complete the life cycle. Because the unembryonated (not infectious) eggs are encapsulated in the liver parenchyma [9] and require a passage through the environment to become embryonated (infectious) [11], the nematode transmission relies on the environmental egg release following the death-decomposition or the ingestion–digestion–excretion process by cannibalism, predation or scavenger [7]. In cities, dogs increase the risk of spreading of *C. hepaticum* by depositing their excrement in the streets or even in green spaces. Stray cats are even more often carriers of *C. hepaticum* [12]. 

*C. hepatica* has a low host specificity, where more than 140 mammal species including humans, dogs, cats, and horses can be infested [11]. However urban rats are generally believed to be the most typical host [11]. This can be explained by its group lifestyle as well as the presence of nests promoting cannibalistic, necrophagic and coprophagic behaviours lead to the circulation of this parasitosis in rat populations. 

In France, epidemiological data on rat capillariasis are lacking. The infection frequency of capillariasis herein we reported in brown rats from the city-centre of Marseille (70%; 14/20) was higher than that we previously reported (41%; 25/61) [13]. Though few rat specimens were herein tested, which represent a limitation of the study, we cannot conclude about changing in the prevalence of this parasitosis. In other large cities, the prevalence in the urban rat population is variable, for example: 88% (176/201) in Baltimore (USA), 83% (337/402) in Salvador de Bahia (Brazil), 45% (33/74) in Rio de Janeiro (Brazil), 36% (241/671) in Vancouver (Canada), 26% (109/422) in Henan Province (China), 22% (21/97) in Kuala Lumpur (Malaysia), 20% (51/254) in Medellin (Colombia), 17% (17/100) in Barcelona (Spain) and 6% (18/302) in Belgrade (Serbia) [14,15,16,17,18,19,20,21,22,23]. 

The description of human cases of hepatic capillariasis is very rare. In Europe, human cases of capillariasis were described only in the Czech Republic [11]. Currently, human cases are mostly reported in Brazil and especially in Asia (China, India, Korea, Philippines, etc.) [24]. The transverse mode of transmission of the parasite to man is exactly the same as between two mammals, namely the ingestion of embryonic eggs present in the environment (especially on the ground). Precarious hygienic conditions in an environment with a high rodent density are important risk factors for transmission to humans. Children are particularly at risk because they are used to putting their hands or objects in general, in their mouths. It is a rare disease, with less than a hundred cases (60% of which affect children under the age of eight) reported worldwide [11]. It is a serious and sometimes fatal human parasitosis which is diagnosed during autopsies. This differential diagnosis should be mentioned during the triad: persistent fever, hepatomegaly and eosinophilic leucocytosis. Until recently, the only diagnostic method to detect *C. hepaticum* was histological analysis of liver biopsies. PCR (18S rRNA gene) is now achievable in specialised laboratories [25]. However, reliable serological tests are not commercialised [26]. Seropositivity does not necessarily certify hepatic parasitism because the ingestion of non-embryonic eggs can also stimulate the production of specific antibodies. Several serological surveys have been carried out as part of epidemiological research in humans living in the same environment as infested rats. In Brazil, for example, the seroprevalence was 1.8% (9/490) [27]. 

In addition, these urban rats are very often infested with *C. hepaticum*. Our aim is to raise awareness among local health authorities of the transmission risk to humans (particularly children and homeless people) of this parasitosis, which remains underestimated and neglected. Prevention should be strengthened in two fields: physicians should be more aware of this neglected disease and rodent-control plans should be promoted by veterinarians and ecologists in all big cities. It will include a reinforcement of the means implemented to maintain the cleanliness of the streets (evacuation of food waste and dog excrement). The population, well informed, should participate in the implementation of these public health measures. Therefore, despite the proven dynamics of *C. hepaticum* life cycle, again supported by our observations, an ecological balance between humans and urban rats will limit the zoonotic risk.

## References

[1] United Nations Human Settlements Programme (UN-Habitat) (2016). World Cities Report 2016: Urbanization and Development: Emerging Futures.

[2] Himsworth C.G., Jardine C.M., Parsons K.L., Feng A.Y.T., Patrick D.M. (2014). The characteristics of wild rat (*Rattus* spp.) populations from an inner-city neighborhood with a focus on factors critical to the understanding of rat-associated zoonoses. PLoS ONE.

[3] Himsworth C.G., Parsons K.L., Jardine C., Patrick D.M. (2013). Rats, cities, people, and pathogens: A systematic review and narrative synthesis of literature regarding the ecology of rat-associated zoonoses in urban centers. Vector-Borne Zoonotic Dis..

[4] Heuser E., Fischer S., Ryll R., Mayer-Scholl A., Hoffmann D., Spahr C., Imholt C., Alfa D.M., Fröhlich A., Lüschow D. (2017). Survey for zoonotic pathogens in Norway rat populations from Europe. Pest. Manag. Sci..

[5] Strand T.M., Lundkvist Å. (2019). Rat-borne diseases at the horizon. A systematic review on infectious agents carried by rats in Europe 1995–2016. Infect. Ecol. Epidemiol..

[6] Moravec F. (1982). Proposal of a new systematic arrangement of nematodes of the family Capillariidae. Folia Parasitol.

[7] Spratt D.M., Singleton G.R., Samuel W.M., Pybus M.J., Kocan A.A. (2001). Hepatic capillariasis. Parasitic Diseases of Wild Animals.

[8] Min B.H., Lee H.S., Kim S.J., Joo K.H. (2013). Ultrastructure of *Capillaria hepatica* (Syn. *Calodium hepatica*) isolated from the liver of mouse infected with artificially embryonated eggs collected from house rats (*Rattus norvegicus*). Appl. Microsc..

[9] Sinniah B., Narasiman M., Habib S., Gaik Bei O. (2014). Prevalence of *Calodium hepaticum* and *Cysticercus fasciolaris* in urban rats and their histopathological reaction in the livers. J. Vet. Med..

[10] Jeong W.I., Do S.H., Hong I.H., Ji A.R., Park J.K., Ki M.R., Park S.C., Jeong K.S. (2008). Macrophages, myofibroblasts and mast cells in a rat liver infected with *Capillaria hepatica*. J. Vet. Sci..

[11] Fuehrer H.P. (2014). An overview of the host spectrum and distribution of *Calodium hepaticum* (Syn. *Capillaria hepatica*): Part 2-Mammalia (excluding Muroidea). Parasitol. Res..

[12] Quadros R.M., Weiss P.H., Miletti L.C., Moura A.B. (2016). Occurrence of *Calodium hepaticum* (Bancroft, 1893) Moravec, 1982 eggs in feces of dogs and cats in Lages, Santa Catarina, Brazil. Rev. Inst. Med. Trop. Sao Paulo.

[13] Davoust B., Boni M., Branquet D., Ducos de Lahitte J., Martet G. (1997). Recherche de trois infestations parasitaires chez des rats capturés à Marseille: évaluation du risque zoonosique [Research on three parasitic infestations in rats captured in Marseille: Evaluation of the zoonotic risk]. Bull. Acad. Natl. Med..

[14] Easterbrook J.D., Kaplan J.B., Vanasco N.B., Reeves W.K., Purcell R.H., Kosoy M.Y., Glass G.E., Watson J., Klein S.L. (2007). A survey of zoonotic pathogens carried by Norway rats in Baltimore, Maryland, USA. Epidemiol. Infect..

[15] Walker R., Carvalho-Pereira T., Serrano S., Pedra G., Hacker K., Taylor J., Minter A., Pertile A., Panti-May A., Carvalho M. (2017). Factors affecting carriage and intensity of infection of *Calodium hepaticum* within Norway rats (*Rattus norvegicus*) from an urban slum environment in Salvador, Brazil. Epidemiol. Infect..

[16] Simões R.O., Luque J.L., Faro M.J., Motta E., Maldonado A. (2014). Prevalência de *Calodium hepaticum* (Sin. *Capillaria hepatica*) em *Rattus norvegicus* em area urbana do Rio de Janeiro, Brasil. Rev. Inst. Med. Trop. Sao Paulo.

[17] Rothenburger J.L., Himsworth C.G., Chang V., LeJeune M., Leighton F.A. (2014). *Capillaria hepatica* in wild Norway rats (*Rattus norvegicus*) from Vancouver, Canada. J. Wildl. Dis..

[18] Wang Z., Lin X., Wang Y., Cui J. (2013). The emerging but neglected hepatic capillariasis in China. Asian Pac. J. Trop. Biomed..

[19] Paramasvaran S., Sani R.A., Hassan L., Krishnasamy M., Jeffery J., Oothuman P., Salleh I., Lim K.H. (2009). Endo-parasite fauna of rodents caught in five wet markets in Kuala Lumpur and its potential zoonotic implications. Trop. Biomed..

[20] Duque B.A., Aranzazu D., Agudelo-Flórez P., Londoño A.F., Quiroz V.H., Rodas J.D. (2012). *Rattus norvegicus* como indicador de la circulación de *Capillaria hepatica* y *Taenia taeniaeformis* en la Plaza Minorista de Medellín, Colombia. Biomedica.

[21] Galán-Puchades M.T., Sanxis-Furió J., Pascual J., Bueno-Marí R., Franco S., Peracho V., Montalvo T., Fuentes M.V. (2018). First survey on zoonotic helminthosis in urban brown rats (*Rattus norvegicus*) in Spain and associated public health considerations. Vet. Parasitol..

[22] Kataranovski D., Kataranovski M., Deljanin I. (2010). Helminth fauna of *Rattus norvegicus* Berkenhout, 1769 from the Belgrade area, Serbia. Arch. Biol. Sci..

[23] Gliga D.S., Pisanu B., Walzer C., Desvars-Larrive A. (2020). Helminths of urban rats in developed countries: A systematic review to identify research gaps. Parasitol. Res..

[24] Fuehrer H.P., Igel P., Auer H. (2011). *Capillaria hepatica* in man—an overview of hepatic capillariosis and spurious infections. Parasitol. Res..

[25] Zhou S.M., Jin X.L., Wang H., Luo H.T., Jia X.S. (2020). Identification and investigation of *Calodium hepaticum* in rodents and insectivores from wuhan section of the Yangtze river in China. Asian Pac. J. Trop. Biomed..

[26] Assis B.C.A., Cunha L.M., Baptista A.P., Andrade Z.A. (2004). A Contribution to the diagnosis of *Capillaria hepatica* infection by indirect immunofluorescence test. Mem. Instit. Oswaldo Cruz.

[27] Rocha E.J., Basano S.A., Souza M.M., Honda E.R., Castro M.B., Colodel E.M., da Silva J.C., Barros L.P., Rodrigues E.S., Camargo L.M. (2015). Study of the prevalence of *Capillaria hepatica* in humans and rodents in an urban area of the city of Porto Velho, Rondônia, Brazil. Rev. Inst. Med. Trop. Sao Paulo.

